# Data-Driven Inverse Design of Silver Nanoparticle Size for Controlled Synthesis Across Multiple Systems Using Conditional Generative Models

**DOI:** 10.3390/ma19091814

**Published:** 2026-04-29

**Authors:** Xingfa Zi, Hongbin Yang, Min Wang, Deqing Zhang, Jun Zeng, Yongan Yang, Youwei Song, Qin Wang, Feiyi Liu

**Affiliations:** 1 School of Physics, Electrical and Energy Engineering, Chuxiong Normal University, Chuxiong 675000, China; zxf@cxtc.edu.cn (X.Z.); wangminyn@163.com (M.W.); cxzdq@cxtc.edu.cn (D.Z.); yya428@cxtc.edu.cn (Y.Y.); fyliu@cxtc.edu.cn (F.L.); 2 Yunnan Tin New Material Company Limited, Kunming 650500, China; 18801068433@163.com (J.Z.); s13973258838@163.com (Y.S.); 18221662016@163.com (Q.W.)

**Keywords:** silver nanoparticles, inverse design, generative models, deep learning, controlled synthesis, process optimization, data-driven optimization

## Abstract

This study aims to develop and systematically evaluate a data-driven inverse-design framework for determining silver nanoparticle (AgNP) synthesis conditions that achieve a prescribed particle size. To this end, a forward surrogate model is first developed to learn the nonlinear mapping from synthesis parameters to particle size, and it is then coupled with target-conditioned inverse models, including conditional generative adversarial network (cGAN), conditional Wasserstein GAN (cWGAN), conditional Wasserstein GAN with gradient penalty (cWGAN-GP), Wasserstein GAN with gradient penalty (WGAN-GP), and other comparable frameworks, to generate feasible synthesis conditions. Three AgNP datasets covering microfluidic and chemical synthesis routes are used for evaluation, and the models are assessed using both experimentally observed target sizes and constructed targets spanning the attainable output range. The results show that conditional adversarial models generally outperform the non-adversarial baselines. Among them, cWGAN shows the most consistent performance across the three datasets, while cGAN remains competitive in the more difficult inverse-design cases. The proposed framework also captures the one-to-many nature of inverse design by producing multiple candidate synthesis conditions for a single target size. In addition, prediction errors increase near the lower and upper boundaries of the feasible size interval. Inverse design is therefore more challenging near these boundaries, although the main comparative conclusions remain unchanged under stricter validation. These findings support the use of forward-constrained conditional generative modeling for target-oriented AgNP synthesis design in limited-data settings.

## 1. Introduction

Silver nanoparticles (AgNPs) have been widely studied because of their distinctive optical, electrical, catalytic, and antibacterial properties, which enable applications in biomedicine, conductive materials, and advanced manufacturing [1,2,3]. In these applications, particle size is a key descriptor because it strongly affects surface activity, stability, and functional performance. As a result, the controllable synthesis of AgNPs with desired sizes is of considerable scientific and practical importance [4,5,6]. However, the size of AgNPs is typically governed by multiple interacting synthesis variables, such as precursor concentration, temperature, reaction time, and other factors, leading to a highly nonlinear process–property relationship [7,8].

Conventional optimization of AgNP synthesis conditions largely relies on empirical trial-and-error strategies [9,10]. Although such approaches can be effective in limited settings, they become inefficient and labor-intensive as the number of possible synthesis parameter combinations increases [11]. Recent advances in machine learning have provided new opportunities for data-driven modeling of complex synthesis systems [12,13]. Most existing studies, however, focus on forward prediction, namely, estimating synthesis outcomes such as particle size and other properties from given synthesis conditions [14,15,16,17,18]. While useful for process understanding, forward models do not directly address the inverse-design problem, where one seeks feasible synthesis conditions for a prescribed target size. This inverse mapping is generally more challenging because it is often non-unique, ill-posed, and highly sensitive to data distribution.

Inverse design is particularly relevant to AgNP synthesis because experimental studies are typically goal-oriented, with researchers seeking synthesis conditions that can reliably produce nanoparticles of a desired size. Compared with forward prediction, this paradigm is more directly aligned with practical synthesis planning, as it aims to identify feasible combinations of precursor concentration, temperature, reaction time and other factors for a prescribed target [19]. In recent years, inverse-design strategies have shown increasing promise in nanoparticle and materials research. For example, multi-target machine learning has been used to inversely design nanoparticles under multiple performance constraints [20], and deep neural networks have been applied to establish both forward and inverse mappings in nanoparticle systems [21]. More broadly, machine learning has been recognized as an effective tool for material synthesis planning and property-oriented design [22]. Despite this progress, inverse design for AgNP synthesis remains intrinsically challenging because similar target sizes may correspond to multiple synthesis conditions, while some candidate solutions may be chemically implausible or experimentally unreliable. These difficulties highlight the need for inverse-design frameworks that can generate feasible synthesis conditions in a controllable and reliable manner.

Generative models offer a promising route for inverse design because they can learn the distribution of feasible synthesis parameters and produce diverse candidate solutions [23,24]. In particular, generative adversarial networks (GANs) [25] are well suited to one-to-many design tasks, where multiple parameter combinations may lead to similar target properties [26,27,28]. However, existing studies have not clearly established their suitability for AgNP inverse design in limited-data experimental settings, where generated synthesis conditions must achieve a prescribed particle size while remaining consistent with the underlying synthesis–size relationship. This limitation defines the research problem addressed in the present study. Conditional GANs (cGANs) provide a natural starting point for this task [29]. Although conditioning on target properties improves controllability, it is not sufficient to ensure physical consistency or robust training. These considerations indicate the need for a target-controllable inverse-design framework that also incorporates guidance from the forward process.

The present study develops a forward-constrained inverse-design framework for AgNP synthesis in a limited-data setting by coupling a forward surrogate model with target-conditioned generative models. Within this framework, adversarial and non-adversarial inverse-design models are compared across three AgNP datasets covering both microfluidic and chemical synthesis routes. Their ability to recover experimentally realizable conditions across the attainable particle-size range is then assessed. The forward surrogate captures the nonlinear relationship between synthesis parameters and particle size and provides a consistent basis for inverse-design evaluation. Based on this forward–inverse coupling, we compare cGAN, conditional Wasserstein GAN (cWGAN) [30], conditional Wasserstein GAN with gradient penalty (cWGAN-GP) [31], and Wasserstein GAN with gradient penalty (WGAN-GP) without target conditioning [32], alongside multilayer perceptron (MLP) and conditional variational autoencoder (cVAE) [33]. This work advances current knowledge of AgNP synthesis and characterization by linking target particle-size specifications to feasible synthesis conditions and clarifying the strengths and limitations of different inverse-design strategies under small-sample conditions.

The remainder of this paper is organized as follows. Section 2 introduces the proposed methodology, including the forward surrogate model and the inverse-design models based on adversarial generation. Section 3 describes the datasets, data preprocessing procedure, target construction strategy, and evaluation metrics. Section 4 presents the results for forward prediction and inverse design under both constructed and experimentally observed target sizes. Section 5 discusses the overall comparative findings, the one-to-many nature of candidate generation, stricter validation analyses and boundary effects across the target space. Finally, Section 6 concludes the study.

## 2. Methodology

In the inverse design of synthesis processes for AgNPs, the fundamental challenge lies in the ill-posed nature of the mapping from target particle size to process parameters [23,26,27,28]. The forward relationship between synthesis conditions and nanoparticle size is governed by complex and highly nonlinear physicochemical mechanisms, including nucleation and growth dynamics [7,8]. Consequently, multiple distinct parameter combinations may lead to similar particle sizes, making the inverse problem inherently one-to-many and difficult to solve directly. In such cases, purely data-driven generative models, such as GAN-based approaches, attempt to learn the inverse mapping implicitly from data. However, without sufficient physical or functional constraints, these models may generate infeasible or physically inconsistent solutions. This issue becomes even more severe under the relatively small sample sizes typical of experimental datasets, where mode collapse and limited generalization are more likely to occur [34].

**Figure 1:**
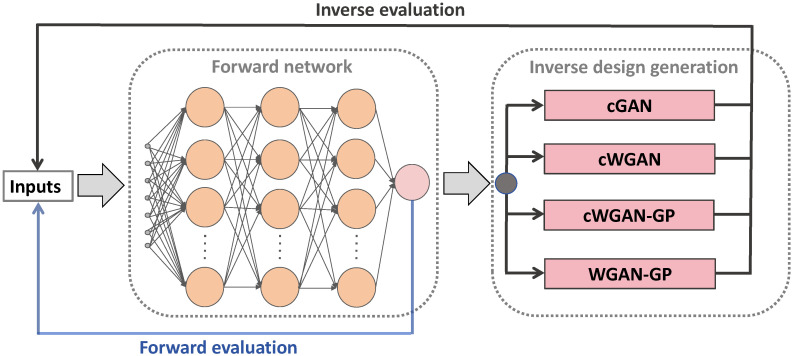
Unified inverse-design architecture of the MLP-based adversarial models, including cGAN, cWGAN, cWGAN-GP, and WGAN-GP.

To address these challenges, we develop a closed-loop inverse-design framework, as illustrated in Figure 1. The overall methodology consists of two tightly coupled components. A forward surrogate network is established to learn the mapping from synthesis parameters to AgNP size and to provide forward evaluation of candidate solutions. Based on this learned relationship, an inverse design module is further constructed to generate synthesis parameters for a prescribed target size through a hierarchy of adversarial models with progressively improved conditioning capability and training stability. In this hierarchy, cGAN is a target-conditioned adversarial model. Both the generator and discriminator receive the desired particle size as input, which improves controllability of the generated synthesis parameters. cWGAN retains this target-conditioning mechanism, but replaces the standard GAN objective with Wasserstein training. As a result, the discriminator is replaced by a critic, and the optimization becomes less sensitive to gradient saturation. WGAN-GP further introduces gradient penalty to enforce the Lipschitz constraint required by Wasserstein training. However, it does not use explicit target conditioning and is therefore used to distinguish the effect of training stabilization from that of target conditioning. cWGAN-GP combines target conditioning, Wasserstein training, and gradient penalty in a single model. It is expected to provide improved controllability together with more stable optimization. The comparison among cGAN, cWGAN, WGAN-GP, and cWGAN-GP helps distinguish the respective contributions of target conditioning, Wasserstein training and gradient-penalty regularization to inverse design performance [29,30,31,32].

For comparison, two non-adversarial inverse-design baselines, namely MLP and cVAE [33], are also included, and their detailed structures are given in Appendix A. Because the available experimental datasets are limited in size, the purpose of this comparison is not to train high-capacity generative models in a data-rich regime. Instead, it is to examine whether target-conditioned generative models can still serve as effective candidate generators under small-data conditions. In addition, these models are evaluated within a framework constrained by the forward process. In this closed-loop workflow, the forward model provides predictive consistency for candidate parameter sets, whereas the inverse generator searches for feasible synthesis conditions that satisfy the target-oriented design objective, both of which are detailed in the following two subsections.

### 2.1. Forward Surrogate Model

The forward surrogate model is constructed to approximate the mapping from synthesis parameters to AgNP size, which can be written as

(1) $$\hat{y}=f_{\theta }\left(x\right),$$

where $x\in R^{d}$ denotes the process parameters, and $\hat{y}$ represents the predicted AgNP size. By learning this mapping from available experimental data, the surrogate model can capture the underlying physical relationships in a data-driven manner.

The forward surrogate model constitutes a central component of the proposed inverse design framework. By embedding the learned forward mapping into the optimization process, it renders the ill-posed inverse design task more tractable and reduces the ambiguity inherent in the one-to-many inverse relationship. At the same time, it imposes a consistency constraint on generated samples, requiring them to be not only statistically plausible but also compatible with the learned forward synthesis process. In addition, it provides an auxiliary supervision signal that stabilizes adversarial training, which is particularly important under limited-data conditions.

In this work, the surrogate model is implemented as an MLP with a compact yet expressive architecture. The network projects the low-dimensional input features into a higher-dimensional latent space to capture complex nonlinear interactions, and subsequently compresses the learned representation through successive hidden layers to preserve the most informative features while reducing redundancy. Such a funnel-shaped architecture is well suited to nonlinear regression under limited-data conditions, as it provides sufficient representational capacity while maintaining generalization ability.

Building on this architecture, each hidden layer adopts the LeakyReLU activation function to strengthen the nonlinear modeling capability of the network while alleviating the dead neuron issue [35]. Dropout regularization is further introduced to enhance model robustness and reduce the risk of overfitting under limited-data conditions [36]. The surrogate model is trained by minimizing the mean squared error (MSE) loss $L_{forward}=E\left[{\left(y-\hat{y}\right)}^{2}\right]$, which provides stable optimization behavior and reliable convergence for continuous regression tasks.

Beyond its predictive role, the forward surrogate model serves as a key component of the inverse design framework. For each generated parameter vector $x_{g}$, the surrogate model produces a corresponding size prediction

(2) $${\hat{y}}_{g}=f_{\theta }\left(x_{g}\right).$$

The discrepancy between ${\hat{y}}_{g}$ and the target size $y_{\mathrm{target}}$ is then used to guide the generator toward parameter combinations that are consistent with the desired design objective. In this way, the generative process is explicitly coupled with the learned forward synthesis relationship, thereby improving the reliability and accuracy of inverse design. The forward surrogate model therefore functions not merely as an auxiliary predictor, but as an essential bridge between data-driven generation and process-consistent constraint learning. The specific hyperparameter settings of the forward surrogate model are provided in Appendix A.

### 2.2. Generative Models for Inverse Design

Next, we introduce the generative models used for inverse design. To examine the effects of conditioning and training stabilization, cGAN, cWGAN, cWGAN-GP, and WGAN-GP are considered within a unified framework. Since these models are all derived from the adversarial learning paradigm, the standard GAN formulation is first briefly outlined to provide the necessary background. In the original GAN framework, the generator maps a latent vector z∼N(0,I) to the parameter space,

(3) $$x_{g}=G\left(z\right),$$

while the discriminator outputs the probability that a sample is drawn from the real distribution,

(4) D(x)∈[0,1].

The corresponding adversarial objective is

(5) $$\min_{G}\max_{D}E_{x∼P_{r}}\left[\text{log}D\left(x\right)\right]+E_{z∼P_{z}}\left[\text{log}\left(1-D\left(G\left(z\right)\right)\right)\right].$$

Both the generator and discriminator are implemented as multilayer perceptrons. The generator progressively transforms low-dimensional noise into the synthesis-parameter space, whereas the discriminator maps the input parameters to a scalar output. Although this formulation enables GAN to approximate the real data distribution $P_{r}\left(x\right)$, it does not incorporate target-specific information and therefore cannot directly support inverse design tasks that require synthesis parameters for a prescribed AgNP size.

To enable target-conditioned generation, the GAN framework is extended to cGAN, in which both the generator and discriminator are conditioned on the target size *y*,

(6) $$x_{g}=G\left(z,y\right),\;D\left(x,y\right).$$

The objective then becomes

(7) $$\min_{G}\max_{D}E_{x,y}\left[\text{log}D\left(x,y\right)\right]+E_{z,y}\left[\text{log}\left(1-D\left(G\left(z,y\right),y\right)\right)\right].$$

This conditioning allows the generator to learn a mapping from (z,y) to feasible process parameters, thereby introducing target-directed controllability into the generation process [29]. Further improvement in training stability and distribution matching motivates the transition to Wasserstein-based adversarial formulations.

Accordingly, the Wasserstein GAN framework is adopted and extended to the conditional setting, resulting in cWGAN. In this formulation, the discriminator is replaced by a critic *D* that outputs real-valued scores, D(x,y)∈R [30]. The corresponding Wasserstein objective is written as

(8) $$L_{W}=E_{x_{g}∼P_{g}}\left[D\left(x_{g},y\right)\right]-E_{x∼P_{r}}\left[D\left(x,y\right)\right],$$

which provides smoother gradients and improved optimization stability. In cWGAN, the Lipschitz continuity required by the Wasserstein formulation is typically enforced through weight clipping, ∥w∥ ≤ c, which may restrict the flexibility of the critic and affect optimization effectiveness [30]. To obtain a more stable and expressive conditional formulation, we replace weight clipping with a gradient penalty and construct cWGAN-GP as the primary generative model [31,32]. The gradient penalty term is defined as [32]

(9) $$L_{GP}=\lambda \;E_{\hat{x}}{\left({∥\nabla_{\hat{x}}D\left(\hat{x},y\right)∥}_{2}-1\right)}^{2},$$

where $\hat{x}$ is sampled along the line connecting real and generated samples. The corresponding critic objective is

(10) $$L_{D}=E_{x_{g}∼P_{g}}\left[D\left(x_{g},y\right)\right]-E_{x∼P_{r}}\left[D\left(x,y\right)\right]+L_{GP}.$$

Building on the forward surrogate model introduced in Section 2.1, we further incorporate its prediction into the conditional generative framework to regularize inverse design. For each generated parameter vector $x_{g}=G\left(z,y\right)$, the predicted particle size is given by ${\hat{y}}_{g}=f_{\theta }\left(x_{g}\right)$, and the forward consistency loss is defined as $L_{forward}=E\left[{\left({\hat{y}}_{g}-y\right)}^{2}\right]$. The generator is then optimized using the combined objective

(11) $$L_{G}=-E_{x_{g}∼P_{g}}\left[D\left(x_{g},y\right)\right]+\alpha \;L_{forward},$$

where the adversarial term promotes distributional realism and the forward consistency term encourages the generated parameters to satisfy the prescribed target size. For comparison, an unconditional WGAN-GP model is also considered by removing the target condition from both the generator and the critic while retaining the same Wasserstein objective and gradient penalty [32]. This comparison enables an explicit assessment of the contribution of conditional information to inverse design performance.

Despite the differences in objective formulation, the generative models considered in this study are built upon a shared architectural backbone. MLPs are used throughout, which are well suited to the low-dimensional and tabular characteristics of microfluidic data. Within this unified design, the generator maps latent variables to the synthesis-parameter space through nonlinear transformations, whereas the discriminator or critic encodes the input into scalar outputs for adversarial learning. As summarized in Table 1, the progression from cGAN to cWGAN, cWGAN-GP, and WGAN-GP reflects a systematic refinement in conditioning mechanism, divergence formulation, critic output, and Lipschitz constraint enforcement. Accordingly, all models share the same data preprocessing procedure and overall backbone design, while differing mainly in whether target conditioning is incorporated, whether the adversarial objective follows the conventional GAN loss or the Wasserstein formulation, and whether the Lipschitz constraint is handled through weight clipping or gradient penalty. Range and diversity regularizations are applied consistently across models, and the resulting framework is therefore well suited to inverse design in microfluidic nanoparticle synthesis under limited-data conditions. Detailed framework descriptions and hyperparameter settings are provided in Appendix A.

## 3. Data and Evaluation Framework

A consistent data foundation is essential for both forward prediction and inverse design in AgNP synthesis. The following parts of this section therefore describe the experimental datasets considered in this study, the preprocessing operations used to standardize the data for model training, and the evaluation protocol adopted to assess performance in both surrogate prediction and inverse design. These steps together ensure the reliability, comparability, and physical interpretability of the subsequent modeling results.

### 3.1. Data

Three experimental datasets on AgNP synthesis are collected from previously published studies and used in this work. Although all three datasets follow the same supervised-regression format, they originate from different experimental routes and therefore differ in sample structure, variable composition, and target-size coverage. Clarifying these differences is important because they directly affect the difficulty of both forward prediction and inverse design. Although all the datasets share a common supervised-regression structure, they differ substantially in synthesis route, variable composition, and scientific focus. This combination of structural consistency and experimental diversity provides a suitable basis for evaluating the proposed framework in both AgNP size prediction and inverse design.

Data1 is derived from the microfluidic AgNP synthesis study of Nathanael et al. [37]. It contains 60 records corresponding to 20 unique operating conditions, with three repeated measurements for each condition. The target variable is particle size, and the input variables are the nucleation constant, growth constant, storage temperature, Dean number, and Reynolds number. These variables mainly describe reaction kinetics and flow behavior in the microfluidic system. Relative to the other two datasets, Data1 covers the broadest target-size interval, from 3.58 to 25.52 nm, and combines repeated observations with a comparatively limited number of unique design points. This structure makes the data scientifically informative, but it also indicates a more heterogeneous inverse-design landscape across the feasible target space.

Data2 is taken from the green-synthesis study of Shabanzadeh et al. using *Vitex negundo* L. extract as both the reducing and stabilizing agent [38]. It contains 30 samples, all of which correspond to unique experimental conditions without replicates. Particle size is again used as the output variable, alongside the molar concentration of AgNO_3_. The weight percentage of *Vitex negundo* extract, reaction temperature, and stirring time are used as inputs. In contrast to Data1, the variables in Data2 are direct operating conditions rather than kinetic or hydrodynamic descriptors. Its target sizes span 15.37–31.79 nm, so the observed distribution is concentrated in the medium-to-large particle-size regime. This gives Data2 a more localized target domain while preserving substantial process variability.

Data3 originates from the montmorillonite-interlayer synthesis study of Shabanzadeh et al. [39]. Like Data2, it consists of 30 unique experimental conditions without replicates. The target variable is AgNP size, and the inputs are AgNO_3_ concentration, reaction temperature, UV–visible wavelength, and montmorillonite interlayer spacing. Compared with Data1 and Data2, Data3 places greater emphasis on structural and characterization-related variables in addition to conventional synthesis settings. Its target-size range is much narrower, from 3.82 to 8.71 nm, meaning that the observed outputs are concentrated in a compact low-size regime.

Despite these differences, the three datasets share a common tabular structure in which experimental conditions are used as inputs and AgNP size is used as the output label, enabling their integration into a unified machine-learning framework. Their different origins and target distributions also help explain part of the performance gap reported later. Data1 is expected to be the most difficult because it combines the widest target interval with only 20 unique conditions. Data2 occupies an intermediate position with a more localized but still broad target domain. Data3 is expected to be comparatively easier because its feasible output space is much narrower and more compact. Their main characteristics are summarized in Table 2.

### 3.2. Data Preprocessing

On the basis of the dataset structure described above, each dataset is standardized into a unified tabular representation for training the forward surrogate model and the adversarial inverse-design frameworks considered in this study. The process variables are treated as input features, and AgNP size is defined as the regression target. After consistency checks and format conversion, the observations are re-indexed to obtain a clean and contiguous data structure.

To support both forward prediction and inverse design, the processed data are further normalized according to the requirements of different model components. The input features and target variable are standardized separately for training the forward surrogate model, while a standardized parameter space is constructed for the adversarial models. In the conditional setting, the target variable is additionally transformed into a normalized condition vector to ensure stable generation [29]. The data are then divided into training and test subsets using an 80%/20% split with a fixed random seed. The lower and upper bounds of each input feature are also retained for projecting generated samples into the admissible physical domain. For the microfluidic dataset, the storage-temperature variable is further constrained during post-processing to maintain consistency with the original experimental setting.

**Figure 2:**
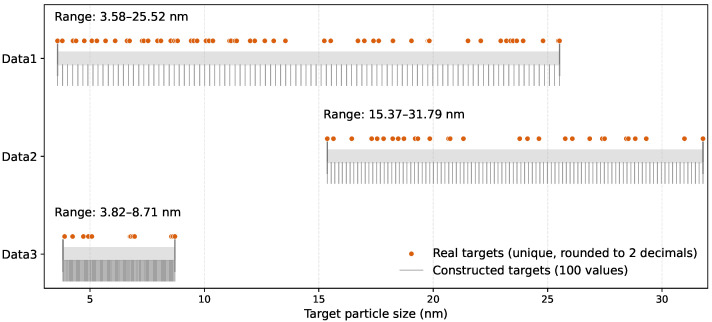
Observed and constructed target-size coverage for Data1, Data2, and Data3.

To enable a rigorous evaluation of inverse design, two complementary target-size sets are prepared. One consists of the unique experimentally observed particle sizes rounded to two decimal places and serves to evaluate the model’s capacity to recover feasible synthesis conditions corresponding to real observations. The other consists of 100 constructed target values spanning the observed particle-size range and provides a denser and more uniform basis for evaluating interpolation capability and target controllability over the feasible output domain. As shown in Figure 2, the experimentally observed targets are sparsely distributed within the size interval of each dataset, whereas the constructed targets provide nearly uniform coverage across the same range.

The 100 constructed targets are generated directly from the minimum and maximum particle sizes in each processed dataset. Let $y_{\min}$ and $y_{\max}$ denote the minimum and maximum values of the target variable. A linearly spaced sequence of 100 values is generated over the interval $\left[y_{\min},y_{\max}\right]$,

(12) $${\tilde{y}}_{i}=y_{\min}+\frac{i-1}{99}\left(y_{\max}-y_{\min}\right),\;i=1,2,\ldots ,100.$$

Each value is then rounded to two decimal places,

(13) $$y_{i}^{\left(100\right)}=\mathrm{round}\left({\tilde{y}}_{i},2\right),\;i=1,2,\ldots ,100.$$

Since rounding may introduce duplicate values, the rounded sequence is deduplicated. If fewer than 100 distinct values remain, an auxiliary candidate pool with a resolution of 0.01 is generated within the same interval,

(14) $$P=\left\{p\;|\;p=\frac{k}{100},\;\left\lfloor 100y_{\min}\right\rfloor \leq k\leq \left\lceil 100y_{\max}\right\rceil \right\}.$$

After restricting this pool to the rounded interval, it is merged with the deduplicated linearly spaced sequence, deduplicated again, and sorted in ascending order. The first 100 distinct values are then retained as the final constructed target set. This procedure preserves the experimentally observed size range while providing approximately uniform coverage of the feasible output domain.

### 3.3. Evaluation

The evaluation framework consists of forward prediction assessment and inverse-design assessment. Forward prediction is used to examine the accuracy of the learned mapping from synthesis conditions to AgNP size and to determine whether the resulting model can serve as a reliable surrogate, whereas inverse-design assessment evaluates the ability of the adversarial models to generate feasible synthesis parameters for prescribed particle-size targets. Since the quality of generated candidates is judged through the forward surrogate, the predictive accuracy of the forward model forms the foundation of the entire evaluation pipeline. As introduced in Section 2.1, the surrogate is implemented as an MLP and optimized using the MSE loss. During training, the model parameters are updated on the training subset, while the loss on the test subset is monitored for early stopping to reduce overfitting [40]. After convergence, the best-performing checkpoint is restored and used to predict both the full dataset and the held-out test set. Predictive performance is quantified in the original particle-size scale using the coefficient of determination ($R^{2}$), mean absolute error (MAE), and root mean squared error (RMSE),

(15) $$\begin{array}{cc} R^{2} & =1-\frac{\sum_{i=1}^{n}{\left({\hat{y}}_{i}-y_{i}\right)}^{2}}{\sum_{i=1}^{n}{\left(y_{i}-\bar{y}\right)}^{2}},\\ \mathrm{MAE} & =\frac{1}{n}\sum_{i=1}^{n}\left|{\hat{y}}_{i}-y_{i}\right|,\\ \mathrm{RMSE} & =\sqrt{\frac{1}{n}\sum_{i=1}^{n}{\left({\hat{y}}_{i}-y_{i}\right)}^{2}}, \end{array}$$

where $y_{i}$ and ${\hat{y}}_{i}$ denote the observed and predicted particle sizes of the *i*th sample, respectively, and $\bar{y}$ is the mean of the observed values. Higher $R^{2}$ values together with lower MAE and RMSE values indicate better predictive performance.

Inverse-design evaluation is carried out after the forward surrogate has been trained and fixed at this stage. The observed target set is used to assess recovery of synthesis conditions associated with real data, whereas the constructed target set is used to evaluate interpolation capability and controllability across the particle-size domain. For a prescribed target particle size $y^{*}$, the adversarial model generates candidate synthesis parameters according to the corresponding generative setting described in Section 2.2. The generated parameters are then mapped back to the physical domain and projected into the admissible range following the preprocessing procedure described in Section 3.2. For the microfluidic dataset, the storage-temperature variable is further discretized during post-processing to maintain consistency with the original synthesis conditions. Each generated candidate parameter set $x_{g}$ is subsequently evaluated by the trained surrogate, and the inverse-design error is defined as $|f_{\theta }\left(x_{g}\right)-y^{*}|$. For a given target, the framework generates 500 candidate parameter sets and retains the top 20 solutions with the smallest absolute errors, thereby enabling assessment of both the best attainable inverse-design accuracy and the stability of high-quality solutions among the leading candidates.

Inverse-design results are summarized at both the target-specific and aggregated levels. For a given target, the evaluation records the best predicted AgNP size, the best absolute error, and the error statistics of the retained top-20 solutions, including the mean, standard deviation, minimum, and maximum values. The mean and standard deviation of the absolute errors over all generated candidates are also calculated to characterize generator quality under each target condition. To quantify the probability of obtaining sufficiently accurate inverse solutions, hit rates under error tolerances of 0.5 nm, 1 nm, 2 nm, and 5 nm are computed as

(16) $$\mathrm{HR}\left(\tau \right)=\frac{1}{N}\sum_{i=1}^{N}1\left(e_{i}\leq \tau \right),$$

where τ denotes the prescribed error tolerance, $e_{i}$ is the absolute error of the *i*th generated candidate, and *N* is the total number of generated candidates for the target under consideration. Since adversarial generators may concentrate solutions near the limits of the admissible domain, the near-boundary ratio is further introduced to evaluate the physical distribution of generated parameters [34]. And it is defined as the proportion of generated feature values located within 5% of the lower or upper bound of the corresponding variable range. The target-wise results are subsequently aggregated through these summary statistics, which together provide a unified evaluation of the inverse-design framework in terms of predictive accuracy, target controllability, robustness, and physical feasibility.

## 4. Results

### 4.1. Forward Prediction

**Figure 3:**
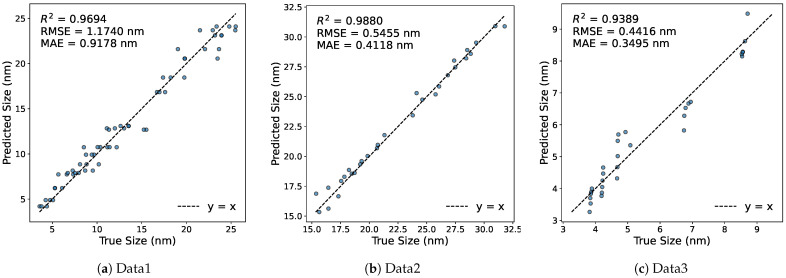
Forward prediction parity plots for the three datasets. The predicted AgNP sizes are plotted against the true values, and the dashed line represents the ideal relation y=x.

In the forward modeling stage, an individual surrogate model is trained for each dataset to approximate the mapping from synthesis parameters to AgNP size. As the same forward surrogate is subsequently used within each dataset for all inverse-design models considered in this study, the corresponding full-sample forward evaluation metrics remain unchanged across those models. Figure 3 presents the parity plots of predicted versus observed AgNP sizes for Data1, Data2, and Data3. The surrogate for Data1 achieves an $R^{2}$ of 0.9694, an RMSE of 1.1740 nm, and an MAE of 0.9178 nm, indicating accurate prediction of particle size from the kinetic and hydrodynamic variables in the microfluidic system. The surrogate for Data2 shows even higher predictive accuracy, with $R^{2}=0.9880$, RMSE =0.5455 nm, and MAE =0.4118 nm, suggesting that the process–size relationship in this dataset is learned with comparatively greater consistency. For Data3, the surrogate yields $R^{2}=0.9389$, RMSE =0.4416 nm, and MAE =0.3495 nm. Although the coefficient of determination is lower than that of the other two datasets, the prediction errors remain small and most data points are still concentrated near the diagonal line.

This result indicates reliable predictive performance in a feature space that includes both synthesis and structural characterization variables. The parity plots are consistent with these quantitative metrics and demonstrate close agreement between predicted and observed AgNP sizes across all three datasets, supporting the use of the trained forward surrogate models in the subsequent inverse-design analysis. In the present study, the forward model is intended to serve as a reliable evaluator within the inverse-design process rather than as an independently optimized predictive model.

### 4.2. Inverse-Design Performance on Constructed Target Particle Sizes

The analysis begins with the constructed target particle sizes because this setting provides a more systematic basis for evaluating inverse-design performance over the feasible target space than can be obtained from experimentally observed targets alone. For this purpose, 100 constructed target particle sizes were introduced to examine model behavior in a continuous target domain. As described Section 3.2 and Section 3.3, these targets provide dense and approximately uniform coverage of the observed particle-size interval, which allows model performance to be examined more systematically across the full range of target values.

Table 3 presents the error-based evaluation on the 100 constructed target particle sizes and reveals substantial differences among the three datasets. This dataset-level ordering is consistent with the data characteristics summarized in Section 3.1. Data1 spans the broadest target interval and has only 20 unique operating conditions despite 60 total records. Data2 occupies an intermediate target domain with one sample per condition. Data3 is confined to a narrow low-size interval. Data1 is the most challenging case, as indicated by the large dispersion of model performance. In this dataset, cVAE achieves the lowest Mean Best Error and Mean TopK Error, with values of 0.1230 and 0.1655, yet its Mean All Error rises to 5.7223, which is much larger than the corresponding values of 0.8227 for cGAN and 0.9118 for cWGAN. This result shows that cVAE can occasionally identify highly accurate candidates, while its generation quality remains unstable across the full candidate set. By contrast, cGAN provides the lowest Mean All Error in Data1, and cWGAN remains highly competitive with a Mean Best Error of 0.1277 and a Mean TopK Error of 0.1661. In Data2, the error magnitudes are much smaller. Although cVAE again attains the lowest Mean Best Error at 0.2144, cWGAN gives the lowest Mean TopK Error and Mean All Error, with values of 0.2290 and 0.4045, which indicates the best balance between top-ranked accuracy and generation stability. Data3 is the most favorable dataset, where cWGAN-GP achieves the lowest Mean Best Error and Mean TopK Error at 0.0870 and 0.0885, while MLP yields the lowest Mean All Error at 0.1485. These results show that the inverse-design difficulty decreases markedly from Data1 to Data3, and that the stronger conditional adversarial models are more effective in maintaining both accuracy and stability across the constructed target space.

Table 4 further clarifies the practical significance of these error statistics by measuring the probability of generating acceptable solutions within fixed tolerances. In Data1, cGAN attains the highest mean hit rate at 0.5 nm and 1 nm, with values of 0.4452 and 0.6730, while cWGAN reaches the highest value of 0.9566 at 2 nm. Although cVAE performs well in Mean Best Error and Mean TopK Error, its hit rates are much lower, reaching only 0.0761 at 0.5 nm and 0.1521 at 1 nm, which confirms that its accurate solutions occur less consistently. In Data2, cWGAN again shows the strongest tolerance-based performance, achieving 0.7783 at 0.5 nm and 0.8930 at 1 nm, while remaining essentially tied with MLP at 2 nm, where both methods reach 0.9700. In Data3, several methods approach saturation in terms of hit-rate performance. MLP records the highest mean hit rate of 0.9200 at 0.5 nm, while cGAN, cWGAN, and cWGAN-GP all achieve nearly perfect hit rates at the 1 nm and 2 nm tolerances. A more detailed discussion of this point is deferred to Section 5.3, where the near-perfect hit rates observed for Data3 are re-evaluated under stricter validation protocols. In combination with the error-based metrics, these results show that low error values do not always translate into stable inverse-design success and further highlight the advantage of cGAN and cWGAN on the more difficult datasets.

**Figure 4:**
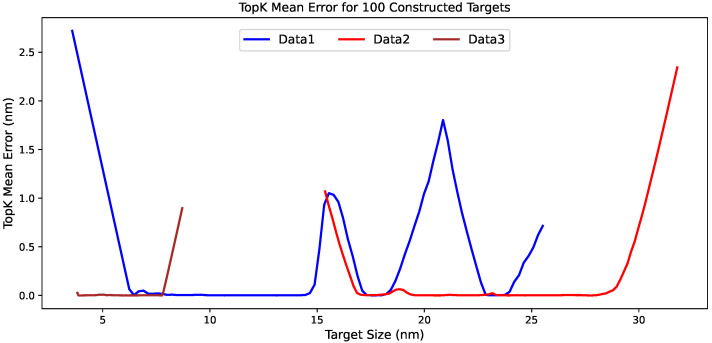
Target-wise TopK mean error curves for the 100 constructed particle-size targets on Data1, Data2, and Data3. The figure shows how the inverse-design difficulty varies across the target range for different datasets.

These tabulated results can be further understood from a target-wise perspective through Figure 4, which illustrates the statistical patterns. The TopK mean error curves show that the inverse-design difficulty is distributed very differently across the three datasets. Data1 contains several separated high-error regions across the target interval, rather than a single concentrated boundary effect, which is consistent with its larger average errors and lower hit rates. Data2 remains much more stable over most of the target range, and the error increases mainly near the upper boundary, a pattern that is consistent with the favorable aggregate metrics and with the remaining difficulty observed for large targets. Data3 shows the smoothest behavior, with TopK mean errors staying close to zero over most of the interval and increasing only slightly near the largest target sizes. This pattern agrees well with the very small error values and the nearly saturated hit rates reported in the two tables. This target-wise pattern indicates that the differences among datasets arise not only from the average quality of the generated candidates, but also from the distribution of inverse-design difficulty across the relevant target space.

The constructed-target evaluation reveals a clear distinction among the three inverse-design scenarios. Data1 is the most sensitive to model choice and requires stronger generative robustness, for which cGAN and cWGAN provide the most reliable performance. Data2 exhibits a more stable inverse relationship, and cWGAN offers the most balanced combination of accuracy, tolerance-based success, and robustness. Data3 is the most well-behaved case, in which several methods already achieve very strong results and the remaining differences mainly reflect the relative emphasis on top-ranked optimality or the compactness of the full predictive distribution. Taken as a whole, these findings indicate that conditional adversarial models provide dependable behavior for continuous target-space inverse design, especially in settings with stronger nonlinearity and less uniform coverage of the feasible region.

### 4.3. Inverse-Design Performance on Experimentally Observed Target Particle Sizes

After analyzing the constructed target particle sizes, we turn to the experimentally observed target particle sizes appearing in the original files and examine inverse-design performance on these targets. In contrast to the denser target-space evaluation of constructed targets, the present analysis focuses on the values directly represented in the available experimental data and therefore provides a complementary view of practical recovery performance.

Table 5 reports the error-based inverse-design results for the experimentally observed target particle sizes. Relative to the constructed targets, the observed targets retain a broadly similar ranking pattern among the models, while showing slightly reduced performance differences in some cases. Data1 remains the most difficult dataset. In this case, cVAE achieves the lowest Mean Best Error at 0.1708, cWGAN-GP gives the lowest Mean TopK Error at 0.2030, and cGAN attains the lowest Mean All Error at 0.7231. At the same time, the Mean All Error of cVAE reaches 4.8698, which is far larger than the corresponding values of cGAN and cWGAN, indicating that its strong best-case accuracy is not accompanied by stable performance across the full candidate set. Data2 shows a more favorable inverse relationship. Although cVAE again gives the lowest Mean Best Error at 0.1907, cWGAN achieves the lowest Mean TopK Error and Mean All Error, with values of 0.2006 and 0.3812, which demonstrate the most balanced performance among the competing methods. In Data3, the best methods become very close. MLP and cWGAN are essentially tied in Mean Best Error and Mean TopK Error at 0.1444 and 0.1448, while MLP gives the lowest Mean All Error at 0.1838. These results suggest that the inverse-design difficulty decreases substantially from Data1 to Data3, and that conditional adversarial models remain more effective than cVAE and WGAN-GP in preserving both accuracy and generation stability on experimentally observed targets.

Table 6 further clarifies the practical meaning of the error statistics by measuring tolerance-based success. In Data1, cGAN achieves the highest mean hit rates at 0.5 nm and 1 nm, reaching 0.5202 and 0.6933, while cWGAN attains the highest value at 2 nm with 0.9507. These results are consistent with the error table and show that cGAN and cWGAN are more reliable than cVAE for generating acceptable solutions at fixed tolerances. In Data2, cWGAN records the highest mean hit rate at 0.5 nm, reaching 0.8254. At 1 nm, MLP gives the highest value of 0.9. At 2 nm, MLP and cWGAN are tied, both reaching 0.9667. This pattern agrees with the error-based results, in which cWGAN provides the strongest balance between top-ranked accuracy and full-sample stability. In Data3, the hit-rate performance of several methods approaches saturation. At 0.5 nm, MLP and cWGAN both achieve 0.8148, and at 1 nm and 2 nm the leading methods are essentially saturated, with MLP, cGAN, and cWGAN all reaching 1 and cWGAN-GP giving 0.9998 and 1. This behavior closely resembles that observed for the constructed target particle sizes, where several leading methods also showed near-saturated hit-rate performance.

**Figure 5:**
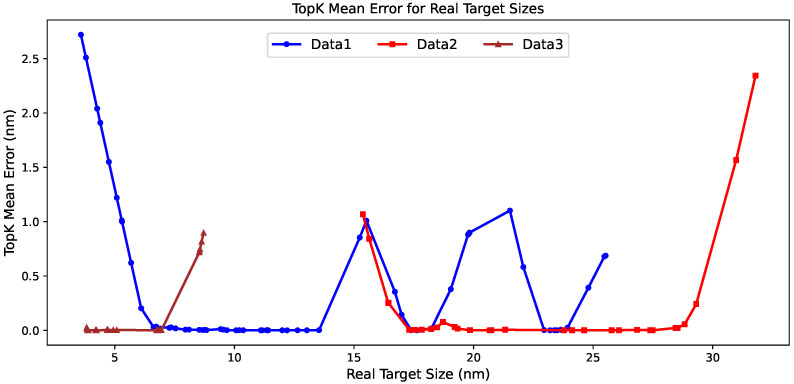
Target-wise TopK mean error curves for the experimentally observed target particle sizes in Data1, Data2, and Data3. The *x*-axis shows the experimentally observed target sizes, and the *y*-axis shows the corresponding TopK mean inverse-design error.

Building on the two tables, Figure 5 provides a target-wise view of the distribution of inverse-design difficulty across the observed target values. Data1 again exhibits several separated high-error regions rather than a single localized peak, which is broadly consistent with the pattern observed for the constructed targets. In the real-target setting, the larger errors remain concentrated in the small-size region around 4 to 6 nm and reappear near 15 to 16 nm, 20 to 22 nm, and 24 to 25 nm, indicating that the difficulty of inverse design in Data1 remains heterogeneous across the target range. Data2 also follows the same general trend as in the constructed-target evaluation, with low errors over most of the observed range and a clear increase near the upper boundary, although an additional local rise is visible around 15 to 16 nm. Data3 remains the smoothest case in both settings, with very small errors across most of the target interval and a noticeable increase only near the upper end of its narrower size range. Taken together, the real-target curves are highly consistent with the constructed-target results in their dataset-level ordering, while also showing that the observed targets reveal a few more localized fluctuations superimposed on the same broad difficulty pattern.

The experimentally observed target evaluation follows the same broad pattern as the constructed-target analysis. Data1 remains the most sensitive to model choice and continues to favor the stronger robustness of cGAN and cWGAN, Data2 again identifies cWGAN as the most balanced method, and Data3 remains the most favorable case with only small differences among the leading models. Compared with the constructed-target results, the ranking pattern is largely unchanged, but the separation among the strongest methods becomes slightly smaller. Both target settings therefore support the same main conclusion that conditional adversarial models provide the most dependable inverse-design behavior for the more difficult datasets, whereas Data3 represents a comparatively well-behaved setting in which model ranking is less decisive.

### 4.4. Experimental Validation of Temperature-Dependent Size Control

To assess the physical plausibility of the size-control tendency captured by the inverse-design framework, we carried out an additional experimental validation for the synthesis of spherical Ag nanoparticles. AgNO_3_ is used as the precursor, Polyvinylpyrrolidone (PVP) as the dispersant, and C_6_H_8_O_6_ as the reducing agent. Under otherwise comparable synthesis conditions, the reaction temperature is varied from 20 to 40 and 60 °C in order to examine its effect on particle size under a common spherical synthesis route [41].

**Figure 6:**
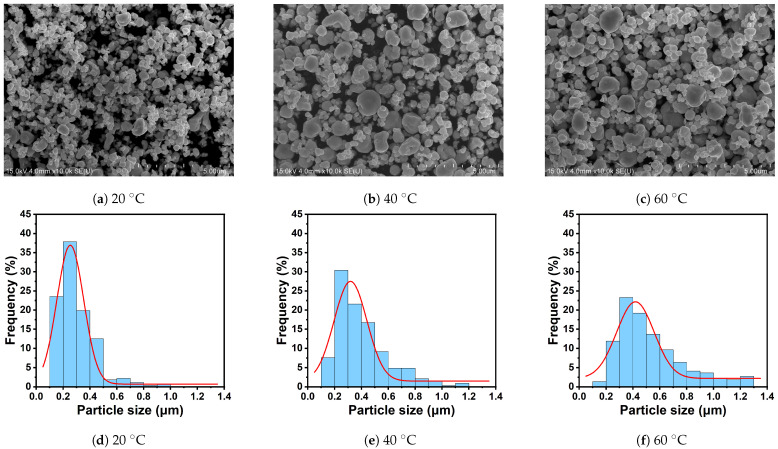
SEM morphologies and particle-size distributions of spherical Ag nanoparticles synthesized at 20, 40, and 60 °C under otherwise comparable conditions. Panels (**a**–**c**) show the SEM images, and panels (**d**–**f**) show the corresponding particle-size distributions. The scale bars in panels (**a**–**c**) are 5.00 μm.

The Scanning Electron Microscope (SEM) images in the top line of Figure 6 show that the products obtained at all three temperatures are dominated by spherical particles, which indicates that the selected synthesis route can reproducibly maintain the target morphology across the investigated temperature range. At the same time, the characteristic particle size increases as the temperature rises. The sample prepared at 20°C contains predominantly smaller particles, whereas the samples obtained at 40 and 60 °C exhibit progressively larger particles and a more evident fraction of coarse particles. This morphological evolution supports the view that temperature acts as an effective control variable for size tuning under otherwise similar experimental conditions.

The particle-size distributions shown in the lower part of Figure 6 support the same trend. At 20 °C, most particles are concentrated approximately in the range of 0.1 to 0.5 μm, with the main peak located near 0.2 to 0.3 μm. At 40 °C, the dominant size range shifts toward larger values, extending to about 0.1 to 0.6 μm, and the distribution center moves closer to 0.3 μm. At 60 °C, the distribution broadens further and shifts again toward larger sizes, with many particles falling between about 0.2 and 0.7 μm and a longer tail extending beyond 1 μm. The concurrent increase in characteristic size and distribution width indicates that the temperature effect is systematic rather than incidental.

These observations provide trend-level experimental support for the physical plausibility of the inverse-design framework. In particular, they show that a synthesis variable identified by the data-driven analysis can indeed drive the particle size in the expected direction while preserving the desired spherical morphology. The present validation is not intended as a pointwise verification of individual inverse-design recommendations. Rather, it is designed to assess the consistency of the learned parameter–size relationship with experimentally observed behavior. From this perspective, the results strengthen the practical relevance of the proposed framework by providing direct experimental support for its inferred size-control tendency. In future work, we will perform more extensive experimental validation and build a more complete database.

## 5. Discussion

### 5.1. Overall Interpretation of Inverse-Design Performance

The results on both constructed and experimentally observed target particle sizes reveal a consistent comparative pattern among the inverse-design algorithms. Across the two evaluation settings, Data1 remains the most challenging dataset, Data2 occupies an intermediate position, and Data3 is the most favorable for inverse design. This ordering is also supported by the dataset origins and distributions described in Section 3.1. Data1 combines the broadest feasible size interval with repeated measurements over only 20 unique conditions. Data2 corresponds to a more localized medium-to-large size regime. Data3 is restricted to a compact low-size interval. At the method level, conditional adversarial models show the strongest robustness across datasets, whereas cVAE is characterized by strong best-case accuracy but limited stability across the full candidate set, and MLP is mainly competitive in the easier setting.

The available data volume is a relevant consideration for the use of generative models. In the present study, this issue is addressed in a task-specific rather than absolute sense. The three datasets are modest in size, so they do not support broad claims about generative modeling in a data-rich setting. However, they remain informative for the inverse-design problem considered here. The input space is low-dimensional and the target variable is one-dimensional. In addition, the practical objective is to generate multiple feasible candidate synthesis conditions for a prescribed particle size rather than to reproduce a highly complex full data distribution. Under this scope, the use of conditional generative models is supported by their conceptual suitability for one-to-many inverse mapping, their consistent comparison against non-generative baselines, and the meaningful performance retained under stricter validation and experimental trend examination. The present results should therefore be interpreted as evidence of feasibility under limited-data conditions rather than as a final demonstration of broad generalizability.

The differences among methods are most pronounced in Data1 and Data2. In Data1, cVAE can achieve very low Mean Best Error values, yet its Mean All Error remains much larger than that of the leading adversarial models, indicating that occasional highly accurate candidates do not necessarily correspond to stable inverse-design quality. By contrast, cGAN and cWGAN perform more reliably, especially under tolerance-based evaluation. In Data2, cWGAN emerges as the most balanced method in both target settings, combining strong error-based performance with consistently favorable hit rates. These results suggest that conditional adversarial models are particularly advantageous once the inverse relationship becomes more nonlinear or heterogeneous [23,24,42].

Among the evaluated algorithms, cWGAN appears to be the most consistently effective method across datasets and evaluation settings. Although it is not always the best model for every individual metric, it repeatedly provides a favorable balance between top-ranked accuracy, full-sample stability, and tolerance-based success. This makes cWGAN the most generally robust choice for inverse design in the present study, while cGAN remains especially competitive in the more difficult setting represented by Data1. More broadly, the comparative results indicate that conditional adversarial models provide the most dependable inverse-design behavior, particularly in cases where the target space is harder to cover uniformly and the inverse mapping is more sensitive to model choice [23,24,42].

### 5.2. One-to-Many Candidate Generation and Its Relevance to Inverse Design

The inverse-design problem considered in this work is inherently one-to-many. A desired nanoparticle size does not necessarily correspond to a unique combination of synthesis conditions. Instead, multiple feasible process-parameter sets may lead to the same target property or to nearly identical target values. This characteristic is particularly important in materials synthesis, where the forward mapping from synthesis conditions to material properties is often many-to-one, and the corresponding inverse mapping is therefore non-unique.

**Figure 7:**
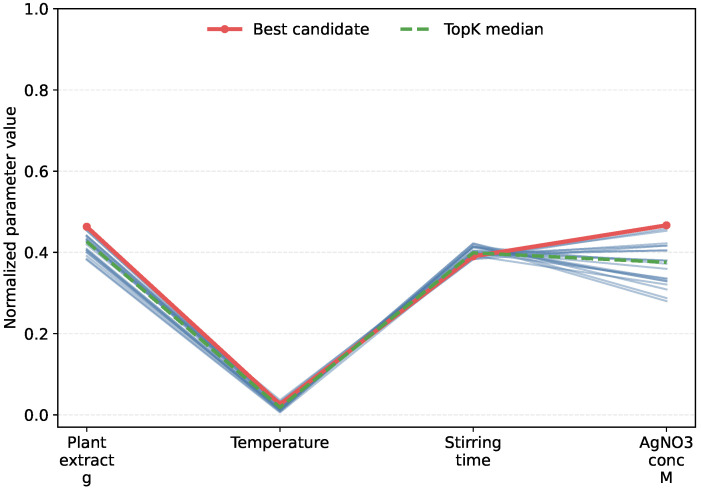
Top-20 candidates generated by cGAN for the Data2 target size of 21.32 nm in normalized process-parameter space. The light-blue lines represent individual candidate parameter sets among the Top-20 generated solutions, with each polyline corresponding to one feasible combination of synthesis parameters. The red solid line denotes the best candidate, and the green dashed line denotes the median profile of the Top-20 candidates.

Figure 7 shows a representative example of this behavior for a Data2 target size of 21.32 nm. The figure presents the Top-20 candidates generated by cGAN in the normalized process-parameter space. Although these candidates correspond to nearly identical target sizes, they remain distributed across different regions of the parameter space. The associated mean absolute error is only 0.005 nm, and the error range is 0.001 to 0.011 nm. This result indicates that the same desired nanoparticle size can be achieved by more than one feasible combination of synthesis parameters, which directly reflects the non-unique nature of the inverse mapping.

This observation helps explain the suitability of cGAN and related generative models for inverse design. A deterministic inverse model that returns only one solution may miss other valid synthesis routes, whereas a generative model can provide multiple alternative candidates for the same target. Such diversity is practically valuable because it gives experimental researchers greater flexibility in selecting synthesis conditions according to additional considerations, including reagent availability, operational simplicity, safety, and cost. Its importance lies not only in the presence of multiple candidates, but also in the fact that diversity is accompanied by high accuracy. The generated solutions are not arbitrary variations in parameter space, but multiple feasible and high-quality candidates associated with the same target. In this sense, the one-to-many generation capability of conditional generative models provides a direct explanation for its effectiveness in inverse design.

The generalizability of the present framework should be understood at the methodological level. The framework is not limited to a specific AgNP chemistry. Instead, it is built on a more general process–property learning structure, where a forward model approximates synthesis outcomes and a conditional inverse generator proposes candidate conditions for prescribed targets. On this basis, the same approach can in principle be extended to other nanoparticle systems, provided that sufficiently informative and consistently measured datasets are available. The framework may also be adapted to systems with different particle morphologies. This would require the conditioning target to include appropriate morphology-related descriptors, such as shape category, aspect ratio, or a joint representation of size and morphology. At present, however, the framework has been validated only for AgNP size design. Its applicability to other nanoparticle types and morphology-controlled systems should therefore be regarded as a promising direction for future work rather than as a result established in the current study.

### 5.3. Robustness of Data3 Under Stricter Validation

The preceding results on both constructed and experimentally observed target particle sizes show that Data3 is consistently the most favorable dataset for inverse design. This unusually strong performance calls for additional scrutiny, since hit rates close to or equal to 1 may raise concerns about overfitting or evaluation optimism [43]. In the original evaluation, Data3 appears markedly easier than the other datasets, with several methods achieving near-saturated hit rates together with low error values. On their own, such results could suggest either that the inverse-design task for Data3 is comparatively easy or that the original evaluation is not sufficiently stringent.

To examine this issue more carefully, we introduced two stricter validation protocols for Data3. One adopted an outer 5-fold external evaluation, in which each fold retained a blind test subset and all preprocessing was fitted only on the corresponding inner training data. The other used a no-leakage double-forward setting, in which candidate ranking and external re-evaluation were performed by two different forward models. These two designs were intended to reduce possible information leakage and to assess the stability of inverse-design performance under more demanding external assessment [43,44]. The corresponding comparison is summarized in Table 7.

The stricter validations led to a noticeable reduction in performance relative to the original evaluation, but the inverse-design results remained clearly effective rather than collapsing. For example, the Mean Best Error increased from 0.1456 in the original evaluation to 0.3878 under the no-leakage double-forward setting and to 0.4127±0.1235 under strict 5-fold external validation. A similar pattern appears in Mean TopK Error, which rose from 0.1476 to 0.3653 and then to 0.4028±0.1188. The hit-rate results also became less saturated, with Hit@1.0 nm decreasing from 1.0000 to 0.8146 in the no-leakage double-forward evaluation and remaining at 0.9073±0.0760 under the strict external protocol. Even so, the model still retained sub-nanometer Best and TopK errors together with comparatively strong tolerance-based success. The external forward surrogates used in these protocols also remained realistic rather than unrealistically perfect, which indicates that the retained inverse-design accuracy cannot be explained simply by a permissive evaluator.

These observations show that the strong Data3 performance is not solely an artifact of leakage, although the original validation likely overestimates the absolute level of performance to some degree. Data3 can therefore be regarded as a particularly sensitive benchmark for validation design, because its target space is narrow and concentrated in the small-size regime, making the task more vulnerable to evaluation leakage and surrogate optimism. At the same time, the stricter assessments support the view that Data3 has a comparatively well-behaved inverse-design landscape and that the learned mapping retains practical predictive value under more realistic external validation. This result strengthens the interpretation of the earlier sections while also highlighting the importance of strict validation protocols in assessing inverse-design models.

### 5.4. Boundary-Effect Analysis Across the Target Space

An additional pattern revealed by the inverse-design results is the presence of a boundary effect across the feasible target interval. The target-wise analyses in the previous subsection show that inverse-design difficulty is not distributed uniformly over the target space. Instead, targets near the lower or upper ends of the attainable particle-size interval tend to be harder to realize accurately than targets in the interior region [45]. To quantify this pattern, the target interval of each dataset was divided into a boundary region and an interior region. The boundary region was defined as the lower and upper 10% of the feasible target interval, while the interior region corresponded to the middle 80%. The resulting quantitative comparison is summarized in Table 8.

The results show that the mean TopK error in the boundary region is consistently higher than that in the interior region for all three datasets. In Data1, the boundary and interior errors are 1.0103 and 0.3017, giving a boundary-to-interior ratio of 3.3492. The contrast becomes much stronger in Data2, where the corresponding errors are 1.0688 and 0.0492, with a ratio of 21.7222. Data3 shows the same tendency, with errors of 0.3387 and 0.0263 and a ratio of 12.8932. This pattern indicates that inverse design becomes substantially more difficult as the target approaches the lower or upper limit of the attainable range. This difficulty is likely related to both the physical constraints of the attainable target range and the reduced support provided by the available training data near the extremes. The same conclusion is supported by the positive association between target-wise TopK error and target-boundary proximity in all three datasets, with Spearman correlations of 0.4347, 0.7616, and 0.5123 for Data1, Data2, and Data3, respectively. Although the strength of the relationship varies across datasets, the directional trend remains consistent, showing that the deterioration near the target-space boundaries is systematic rather than incidental.

An additional issue is the origin of this boundary effect. The current evidence does not support a purely model-induced explanation. If the effects are driven mainly by a geometric tendency of the generator to place candidates near the boundaries of parameter space [34], a similar positive relationship between error and the near-boundary ratio of generated parameters should appear across all three datasets. In practice, this pattern is not observed consistently. A positive association is found in Data1 and Data2, but it weakens and becomes negative in Data3. This result suggests that concentration of generated solutions near parameter-space boundaries may contribute to reconstruction difficulty in some cases, but it cannot fully account for the pattern observed across datasets.

The observed boundary effect is therefore more appropriately interpreted as a combined consequence of the physical system and the learning setting. Near the lower and upper limits of the feasible particle size interval, the inverse mapping is more constrained and more sensitive. Under these conditions, the synthesis parameters that can accurately realize the target become fewer and less robust. Meanwhile, the available training data provide weaker support in these extreme regions, which increases the difficulty of both forward approximation and inverse generation. This pattern suggests that the main signal is associated with the target-space limits of the underlying synthesis system, while data sparsity and finite model capacity further amplify the difficulty near those limits. From a practical perspective, inverse-design recommendations near the extremes of the attainable particle-size interval should be treated more cautiously than those in the interior region.

## 6. Conclusions

In this work, an inverse design framework for AgNP synthesis is established by combining a forward prediction model with conditional generative models. The purpose of this framework is to address the non-unique relationship between synthesis conditions and particle size, where different parameter combinations may lead to similar target outputs. By introducing forward prediction into the inverse design process, the generated synthesis conditions can also be examined in terms of their consistency with the learned process–property relationship.

Three AgNP datasets with different synthesis characteristics are used to evaluate the proposed method. Several models, including MLP, cVAE, cGAN, cWGAN, cWGAN-GP, and WGAN-GP, are compared under the same inverse design task. The results show that conditional adversarial models generally perform better than the other methods. Among them, cWGAN gives the most consistent overall results across the three datasets, while cGAN also shows competitive performance, especially in the more difficult inverse design cases. By contrast, MLP and cVAE can provide reasonable predictions in some cases, but their overall stability and adaptability remain relatively limited.

The results further indicate that inverse design performance depends strongly on the characteristics of the dataset. For target particle sizes located in the central region of the feasible interval, most models perform better than they do near the lower and upper boundaries. This suggests that inverse mapping becomes more difficult in boundary regions because these regions are close to the feasible limits of the target space and are also supported by relatively sparse training data. In addition, the generative models are able to provide multiple candidate synthesis conditions for a given target size, which is consistent with the one-to-many nature of practical synthesis design. A stricter validation on Data3 also shows that the absolute prediction accuracy decreases compared with the original evaluation, but the relative performance ranking of the main models remains largely unchanged.

The present study shows that conditional generative models can be effective for inverse design in AgNP synthesis, particularly for problems involving nonlinear and non-unique process–property relationships. This conclusion should be understood in the context of a limited-data and low-dimensional experimental setting rather than as a broad claim about generative modeling under all conditions. Experimental examination of the generated conditions also supports the main trend predicted by the model, indicating that the inverse design results are physically reasonable to some extent. The proposed framework provides a practical route for estimating synthesis conditions corresponding to a desired particle size.

Its underlying forward–inverse structure is not limited to AgNPs and may be extended to other nanoparticle synthesis systems if suitable datasets are available. It may also be adapted to morphology-aware design by incorporating appropriate target descriptors for particle shape and related structural characteristics. At the same time, the present study validates the framework only for AgNP size design. Broader applicability should therefore be regarded as a promising extension that still requires further verification. Future work should focus on incorporating larger and more diverse datasets, improving the treatment of boundary targets, and extending the current single-target design to multi-objective cases involving particle size, morphology, and other functional properties.

## Figures and Tables

**Table 1:** Comparison of cGAN, cWGAN, cWGAN-GP, and WGAN-GP in this study.

Characteristic	cGAN	cWGAN	cWGAN-GP	WGAN-GP
Conditioning on target *y*	Yes	Yes	Yes	No
Divergence metric	JS divergence	Wasserstein	Wasserstein	Wasserstein
Output of discriminator	Probability	Score	Score	Score
Sigmoid activation	Yes	No	No	No
Lipschitz constraint	No	Weight clipping	Gradient penalty	Gradient penalty
Training stability	Low	Medium	High	High
Mode collapse risk	High	Medium	Low	Low
Physical consistency	Weak	Medium	Strong	Strong

**Table 2:** Summary of the three AgNP synthesis datasets used in this study.

Dataset	Samples	Unique Conditions	Replicates	Target Range (nm)	Main Characteristic
Data1	60	20	3 per condition	3.58–25.52	Microfluidic synthesis
Data2	30	30	None	15.37–31.79	Green synthesis
Data3	30	30	None	3.82–8.71	Montmorillonite synthesis

**Table 3:** Inverse-design performance comparison of different algorithms on constructed target particle sizes for Data1, Data2, and Data3. In this table, Best, TopK, and All correspond to Mean Best Error, Mean TopK Error, and Mean All Error, respectively. Lower values indicate better performance. The optimal value in each column is highlighted in bold.

Model	Data1			Data2			Data3		
	Best	TopK	All	Best	TopK	All	Best	TopK	All
MLP	3.7800	3.8269	4.0047	0.2436	0.2557	0.4580	0.0904	0.0936	**0.1485**
cVAE	**0.1230**	**0.1655**	5.7223	**0.2144**	0.2405	4.2330	0.0913	0.1307	1.1556
cGAN	0.3853	0.4434	**0.8227**	0.2396	0.2531	0.5217	0.0871	0.0888	0.1908
cWGAN	0.1277	0.1661	0.9118	0.2250	**0.2290**	**0.4045**	0.0930	0.1059	0.2119
cWGAN-GP	0.1281	0.1693	1.3734	0.2306	0.2358	0.5092	**0.0870**	**0.0885**	0.2033
WGAN-GP	0.1326	0.2100	6.7519	0.2185	0.2793	5.6054	0.0885	0.1165	2.0711

**Table 4:** Inverse-design hit-rate comparison of different algorithms on constructed target particle sizes for Data1, Data2, and Data3. Higher values indicate better performance. The optimal value in each column is highlighted in bold.

Model	Data1			Data2			Data3		
	0.5 nm	1 nm	2 nm	0.5 nm	1 nm	2 nm	0.5 nm	1 nm	2 nm
MLP	0.0596	0.1200	0.2370	0.7317	0.8898	**0.9700**	**0.9200**	**1.0000**	**1.0000**
cVAE	0.0761	0.1521	0.2915	0.1002	0.1935	0.3535	0.2305	0.4490	0.8421
cGAN	**0.4452**	**0.6730**	0.9071	0.6404	0.8586	0.9691	0.9099	**1.0000**	**1.0000**
cWGAN	0.3256	0.5647	**0.9566**	**0.7783**	**0.8930**	0.9700	0.9110	1.0000	**1.0000**
cWGAN-GP	0.2715	0.4957	0.7354	0.6384	0.8804	0.9692	0.9059	0.9999	**1.0000**
WGAN-GP	0.0444	0.0897	0.1785	0.0603	0.1207	0.2304	0.1407	0.2863	0.5204

**Table 5:** Inverse-design performance comparison of different algorithms on the experimentally observed target particle sizes in the original files for Data1, Data2, and Data3. In this table, Best, TopK, and All correspond to Mean Best Error, Mean TopK Error, and Mean All Error, respectively. Lower values indicate better performance. The optimal value in each column is highlighted in bold.

Model	Data1			Data2			Data3		
	Best	TopK	All	Best	TopK	All	Best	TopK	All
MLP	3.7451	3.7816	3.9291	0.2113	0.2232	0.4199	0.1444	0.1448	**0.1838**
cVAE	**0.1708**	0.2089	4.8698	**0.1907**	0.2216	4.7981	0.1474	0.1801	1.6670
cGAN	0.3866	0.4121	**0.7231**	0.2150	0.2291	0.5006	0.1456	0.1476	0.2465
cWGAN	0.1793	0.2046	0.7931	0.1970	**0.2006**	**0.3812**	**0.1444**	**0.1448**	0.1941
cWGAN-GP	0.1743	**0.2030**	1.1890	0.2000	0.2055	0.4707	0.1447	0.1456	0.2342
WGAN-GP	0.1834	0.2726	6.9983	0.1946	0.2517	5.6028	0.1464	0.1719	2.0004

**Table 6:** Inverse-design hit-rate comparison of different algorithms on the experimentally observed target particle sizes in the original files for Data1, Data2, and Data3. Higher values indicate better performance. The optimal value in each column is highlighted in bold.

Model	Data1			Data2			Data3		
	0.5 nm	1 nm	2 nm	0.5 nm	1 nm	2 nm	0.5 nm	1 nm	2 nm
MLP	0.0933	0.1425	0.2586	0.7884	**0.9000**	**0.9667**	**0.8148**	**1.0000**	**1.0000**
cVAE	0.0984	0.1801	0.3605	0.0957	0.1867	0.3355	0.1380	0.2375	0.5011
cGAN	**0.5202**	**0.6933**	0.9407	0.6648	0.8765	0.9666	0.8146	**1.0000**	**1.0000**
cWGAN	0.4124	0.6564	**0.9507**	**0.8254**	0.8990	0.9667	**0.8148**	**1.0000**	**1.0000**
cWGAN-GP	0.3056	0.5691	0.7888	0.7160	0.8952	0.9666	0.8148	0.9998	**1.0000**
WGAN-GP	0.0477	0.0951	0.1864	0.0686	0.1376	0.2637	0.1896	0.3667	0.5367

**Table 7:** Comparison of the original and stricter validation results on Data3 for the real target particle sizes using cGAN. The “Original” row reports the standard evaluation results, the “No-leak double-forward” row reports the external forward-evaluation results without leakage, and the “Strict 5-fold external” row reports the mean ± standard deviation across the outer folds. Lower error values and higher hit rates indicate better performance.

Validation Setting	Mean Best Error (nm)	Mean TopK Error (nm)	Mean All Error (nm)	Hit-Rate at 0.5 nm	Hit-Rate at 1.0 nm
Original	0.1456	0.1476	0.2465	0.8146	1.0000
No-leak double-forward	0.3878	0.3653	0.3521	0.7870	0.8146
Strict 5-fold external	0.4127±0.1235	0.4028±0.1188	0.4282±0.1222	0.7163±0.1188	0.9073±0.0760

**Table 8:** Quantitative analysis of boundary effects based on the 100 constructed targets. The boundary region comprises the lower and upper 10% of the target interval, and the interior region comprises the middle 80%. “Boundary TopK Error” and “Interior TopK Error” are the mean TopK errors in the two regions, and “Boundary/Interior” is their ratio. “ρ(Error, Target–Boundary Proximity)” measures the association between target-wise TopK error and proximity to the target-space boundary, whereas “ρ(Error, Boundary Ratio)” measures the association between target-wise TopK error and the near-boundary ratio of TopK candidates in parameter space. “*p* (Boundary vs. Interior)” gives the significance of the difference between the two regions.

Dataset	Boundary TopK Error	Interior TopK Error	Boundary/ Interior	ρ (Error, Target–Boundary Proximity)	ρ (Error, Boundary Ratio)	*p* (Boundary vs. Interior)
Data1	1.0103	0.3017	3.3492	0.4347	0.5666	0.0001
Data2	1.0688	0.0492	21.7222	0.7616	0.3366	<$10^{-4}$
Data3	0.3387	0.0263	12.8932	0.5123	−0.2361	0.0684

**Figure A1:**
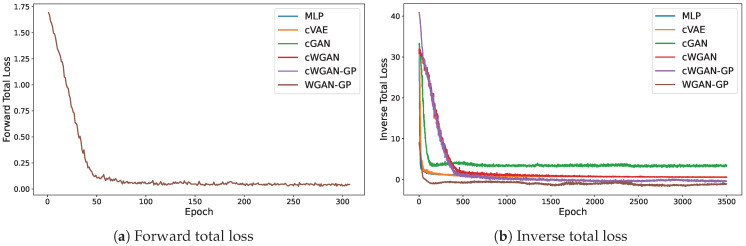
Training loss comparisons for the six inverse-design models.

## Data Availability

The data used in this study are derived from previously published articles. The corresponding sources are Nathanael et al. [37], Shabanzadeh et al. [38], and Shabanzadeh et al. [39].

## Appendix A. Hyperparameter Settings and Algorithmic Configurations

This appendix summarizes the architectures and optimization settings used in this study. A shared forward surrogate model is used to assess the size consistency of generated synthesis conditions. The surrogate is a multilayer perceptron with hidden layers of 64, 32, and 16 neurons, LeakyReLU(0.2) activations, and dropout of 0.1 in the first two hidden layers. The output layer predicts AgNP size. Training minimizes the mean squared error using Adam with a learning rate of $8\times 10^{-4}$, weight decay of $1\times 10^{-4}$, a maximum of 2000 epochs, and early stopping with a patience of 150.

As non-adversarial inverse design baselines, we implement an inverse MLP and a cVAE, both conditioned on target particle size. The inverse MLP maps a one-dimensional target input to the five-dimensional process parameter space through hidden layers of 64, 128, and 64 neurons, followed by a Tanh output layer. It is trained with Adam using a learning rate of $1\times 10^{-3}$ and weight decay of $1\times 10^{-5}$ for up to 3500 epochs, with early stopping based on the test supervised loss. Its objective combines a supervised reconstruction term, a forward consistency term, and a range penalty, with $\lambda_{\mathrm{cons}}=28$ and $\lambda_{\mathrm{range}}=4$. Gaussian perturbations with a standard deviation of 0.03 are added to the standardized target input during candidate generation.

The cVAE captures the non-uniqueness of the inverse mapping through latent variable sampling. It uses a one-dimensional condition and an eight-dimensional latent variable. The encoder consists of hidden layers of 64 and 32 neurons, followed by separate linear layers for the latent mean and log variance. The decoder reconstructs the process parameters through hidden layers of 64, 128, and 64 neurons and a final Tanh output layer. Training uses Adam with a learning rate of $1\times 10^{-3}$ and weight decay of $1\times 10^{-5}$ for 3500 epochs. The loss combines a reconstruction term, a KL regularization term with $\beta_{\mathrm{KL}}=0.01$, a forward consistency term, a range penalty term, and a diversity regularization term weighted by $\lambda_{\mathrm{cons}}=28$, $\lambda_{\mathrm{range}}=4$, and $\lambda_{\mathrm{div}}=0.08$, respectively.

The adversarial inverse design models include cGAN, cWGAN, cWGAN-GP, and WGAN-GP. Specifically, cGAN is the standard conditional GAN used as a target-conditioned adversarial baseline, cWGAN is the conditional Wasserstein variant, cWGAN-GP is the conditional Wasserstein variant with gradient penalty, and WGAN-GP is the non-conditional Wasserstein variant with gradient penalty used to examine the effect of removing explicit target conditioning. The unified workflow of these adversarial inverse design models is summarized in Algorithm A1. All use a generator driven by an eight-dimensional latent noise vector. The generator is a fully connected network with hidden layers of 64, 128, and 64 neurons and a final Tanh output layer. The discriminator or critic uses hidden layers of 128, 64, and 32 neurons and outputs a scalar score. The conditional models additionally take a one-dimensional target variable. cGAN uses a sigmoid discriminator output, whereas cWGAN, cWGAN-GP, and WGAN-GP use linear critic outputs.

All GAN-based inverse-design models, including cGAN, cWGAN, cWGAN-GP, and WGAN-GP, are trained for 3500 epochs. cGAN uses Adam with learning rates of $2\times 10^{-4}$ for both generator and discriminator and β=(0.5,0.999), and its generator objective combines adversarial loss, consistency loss, range penalty, and diversity penalty, with weights of 28, 4, and 0.08 for the latter three terms. cWGAN, cWGAN-GP, and WGAN-GP use a Wasserstein-based framework with generator and critic learning rates of $6\times 10^{-5}$ and $8\times 10^{-5}$, β=(0.5,0.9), five critic updates per generator update, a range penalty weight of 4.0, and a diversity penalty weight of 0.08; cWGAN and cWGAN-GP additionally include a conditional consistency term with weight 28, whereas WGAN-GP omits the explicit condition input and the corresponding consistency term. cWGAN enforces the Lipschitz constraint through weight clipping with a clipping value of 0.01, whereas cWGAN-GP and WGAN-GP use gradient penalty with $\lambda_{gp}=10$. The loss curves in Figure A1 indicate stable optimization behavior. The forward total losses decrease rapidly and are nearly identical across models, indicating consistent training of the shared surrogate, while the inverse total losses also decrease and then stabilize for all methods, demonstrating satisfactory convergence of both non-adversarial and adversarial inverse-design models and supporting the reliability of all six optimization pipelines.

**Algorithm A1** Inverse Design Framework of cGAN, cWGAN, cWGAN-GP, and WGAN-GP
1: **Input:** Dataset $D=\{\left(x_{i},y_{i}\right)\}$, $x\in R^{d}$, y∈R 2: **Parameters:** - Noise dim $d_{z}=8$, generator/critic hidden dims - Critic steps $n_{c}=5$, loss weights $\lambda_{\mathrm{cons}},\lambda_{\mathrm{range}},\lambda_{\mathrm{div}},\lambda_{\mathrm{gp}}$ 3: **procedure** Preprocess(D) 4: Standardize x and *y*, split into train/test 5: **end procedure** 6: **procedure** TrainForwardSurrogate($x_{\mathrm{train}},y_{\mathrm{train}}$) 7: Train MLP $f_{\theta }$ with MSE loss $E[{\left(y-f_{\theta }\left(x\right)\right)}^{2}]$, early stopping, then freeze 8: **end procedure** 9: **procedure** ConsistencyLoss($x_{g}^{\mathrm{phys}},y_{\mathrm{target}}$) 10: ${\hat{y}}_{g}=f_{\theta }\left(x_{g}^{\mathrm{phys}}\right)$, $L_{\mathrm{cons}}=E\left[{\left({\hat{y}}_{g}-y_{\mathrm{target}}\right)}^{2}\right]$ 11: **end procedure** 12: **procedure** RangePenalty($x_{g}^{\mathrm{phys}}$) 13: $L_{\mathrm{range}}=\frac{1}{d}\sum_{j}E\left[\max{\left(0,l_{j}-x_{g,j}\right)}^{2}+\max{\left(0,x_{g,j}-u_{j}\right)}^{2}\right]$ 14: **end procedure** 15: **procedure** DiversityPenalty($z_{1},z_{2}$) 16: $L_{\mathrm{div}}=-\frac{E[|x_{g,1}-x_{g,2}|]}{E[|z_{1}-z_{2}|]+\varepsilon }$ 17: **end procedure** 18: **procedure** GradientPenalty($C,x,x_{g},c$) 19: $\hat{x}=\alpha x+\left(1-\alpha \right)x_{g}$, α∼U(0,1) 20: $L_{\mathrm{gp}}=E[(∥\nabla_{\hat{x}}C\left(\hat{x},c\right){∥}_{2}-1{)}^{2}]$ (or without c) 21: **end procedure** 22: **procedure** TrainAdversarial 23: **for** epoch =1 to $N_{g}$ **do** 24: **if** model = cGAN **then** 25: Update D: $L_{D}=\frac{1}{2}\left[\mathrm{BCE}\left(D\left(x,c\right),1\right)+\mathrm{BCE}\left(D\left(G\left(z,c\right),c\right),0\right)\right]$ 26: Update G: $L_{G}=\mathrm{BCE}\left(D\left(G\left(z,c\right),c\right),1\right)+\lambda_{\mathrm{cons}}L_{\mathrm{cons}}+\lambda_{\mathrm{range}}L_{\mathrm{range}}+\lambda_{\mathrm{div}}L_{\mathrm{div}}$ 27: **else if** model = cWGAN **then** 28: **for** t=1 to $n_{c}$ **do** 29: Update C: $L_{C}=-\left(E\left[C\left(x,c\right)\right]-E\left[C\left(G\left(z,c\right),c\right)\right]\right)$, clip weights 30: **end for** 31: Update G: $L_{G}=-E\left[C\left(G\left(z,c\right),c\right)\right]+\lambda_{\mathrm{cons}}L_{\mathrm{cons}}+\lambda_{\mathrm{range}}L_{\mathrm{range}}+\lambda_{\mathrm{div}}L_{\mathrm{div}}$ 32: **else if** model = cWGAN-GP **then** 33: Same as cWGAN but replace weight clipping with $L_{C}\leftarrow L_{C}+\lambda_{\mathrm{gp}}L_{\mathrm{gp}}$ 34: **else** 35: Same as cWGAN-GP without condition (WGAN-GP); optionally omit $L_{\mathrm{cons}}$ 36: **end if** 37: **end for** 38: **end procedure** 39: **procedure** InverseGeneration($y^{*}$) 40: Standardize $y^{*}$, sample z, generate $x_{g}=G\left(z,c^{*}\right)$ (or G(z)) 41: Map to physical space, predict ${\hat{y}}_{g}=f_{\theta }\left(x_{g}^{\mathrm{phys}}\right)$, rank by $|{\hat{y}}_{g}-y^{*}|$, return top-*K* 42: **end procedure** 43: $\left(x_{\mathrm{train}},y_{\mathrm{train}},x_{\mathrm{test}},y_{\mathrm{test}}\right)\leftarrow \mathrm{Preprocess}\left(D\right)$ 44: $f_{\theta }\leftarrow \mathrm{TrainForwardSurrogate}\left(x_{\mathrm{train}},y_{\mathrm{train}}\right)$ 45: Build generator *G* and discriminator/critic *C* (MLPs) 46: TrainAdversarial() 47: Generate candidates for each target $y^{*}$ via $\mathrm{InverseGeneration}(y^{*})$
